# Mastitis on Rabbit Farms: Prevalence and Risk Factors

**DOI:** 10.3390/ani8060098

**Published:** 2018-06-20

**Authors:** Joan M. Rosell, L. Fernando de la Fuente

**Affiliations:** 1 Cunivet Service. P.O. Box 518, 43080 Tarragona, Spain; 2 Department de Producción Animal, Facultad de Veterinaria, Avda. Profesor Pedro Cármenes s/n, Universidad de León, 24071 León, Spain; f.fuente@unileon.es

**Keywords:** biosecurity, disease prevention, mastitis prevalence, rabbit welfare, risk factor

## Abstract

**Simple Summary:**

Mastitis has been a subject of interest on rabbit farms. To better understand various aspects of this disease, we evaluated the work done during 2001–2017, on 531 farms in Spain and Portugal. We measured the cases of mastitis on the days of visit, with the examination of lactating females. We found an average of 4% of mastitic does; they were mostly chronic forms, since females with acute mastitis often die in a few hours. Several factors affect the occurrence of mastitis. For example, rabbit lines have different susceptibilities. The number of batches per maternity barn also influences the occurrence of disease; therefore, a single batch is better, or the duo system, which consists of moving females near parity to a clean and disinfected room. In the last 200 visited farms, we found that injectable antimicrobials were often used for the mastitis control, in addition to the hygiene practices (for example, culling diseased does and avoiding their kits being fostered).

**Abstract:**

In this cross-sectional study, prevalence of clinical mastitis (PCM) and farm-specific risk factors were determined on 531 doe rabbit farms in Spain and Portugal, from January 2001 through March 2017. The information was obtained by carrying out 2367 visits and doing physical examinations of 144,455 lactating does, sorted in 2635 cohorts. Overall mean PCM was 4.05% (CI_95%_ [3.87–4.22]), (minimum to maximum: 0–36.00% PCM). This study suggests that PCM was influenced by the variable *number of batches* (a batch was a group of does served the same day), per maternity barn (*p* < 0.0001). The *duo system* (does being moved to clean disinfected barns for parturition), was also an enabling risk factor for CM. The *day of service* or *lactation stage* also affected PCM. Lastly, there was an effect of the breeds or lines (*p* < 0.0001); mean PCM ranged between 1.29% and 7.09%. A subset of data obtained from 200 farms visited during January 2012–March 2017, was recorded to describe the use of antimicrobials against mastitis. Changes in host, husbandry, environment, and biosecurity practices, are highlighted to provide health and welfare benefits for breeding rabbits.

## 1. Introduction

On commercial farms housing the European rabbit (*Oryctolagus cuniculus*), a lactating female can produce a total quantity of milk equivalent to her body weight (BW), with a high content of nutrients, except lactose, through 4–5 pairs of mammae, in the course of 35 days [1]. This effort predisposes to disorders affecting the mammae during lactation, mainly intramammary bacterial infection, leading to disease and inflammation, i.e., mastitis [2]. Prevalence of clinical mastitis and risk factors are variables of interest, (a) from the health and welfare standpoint [3]; (b) to improve disease control [4]; (c) in relation to Public Health [5,6]; and (d) because the resulting economic damage is also relevant, mainly with regard to reproduction. For example, fertility in does with clinical mastitis (CM) decreases in relation to apparently healthy ones [7]. Besides, declining milk production and viability in kits is to be expected in mastitic does [8]. In farms with high prevalence of CM (PCM), producers had to increase doe replacements, due to culling [9] and mortality, as a result of an acute form. Lastly, condemnations in the abattoir also had economic significance [10].

Clinical mastitis, caused by natural infections, in the female rabbit, can be either acute or chronic. Acute mastitis can progress to gangrenous necrosis, leading frequently to septicemia and death. Although this condition has been previously reported as “blue breast” [11], we often observed a “red-black” discoloration of the mammary gland (interpreted as coagulative necrosis), in accordance with Miller and Zachary [12]. On-farm mean monthly mortality risk, for mastitis compatible with acute forms, was 0.16% [13]. Mastitis might become chronic, and later on, purulent or purulent-gangrenous; in such cases, with a median monthly culling risk of 0.50% [14]. On the other hand, there might be a complete recovery. The differences in presentation were likely attributable to bacterial virulence factors and host immunity [15].

In Europe, usable information on prevalence of CM in rabbits is scarce. In France, for example, reported mean PCM was 14.3%, on 32 French farms [16]. In a study in Spain, determined PCM was 11.6% on 39 farms, with 1637 examined does from January 1979 to June 1983 [17]. During July 1983–December 1985, 3038 does were examined on 101 farms, with a PCM = 10.6%, and during 1986 through 1996 (103,968 examined does on 762 farms in Spain), PCM was 5.2% [8].

Environmental risk factors enabling infections of the mammae can be (a) traumatic gland and nipple damage caused by suckling kits or occurring in the housing; (b) climatic stress, such as cold air streams; or (c) a combination of moisture plus dirt in the nest [8]. In an earlier study, we determined PCM and risk factors related to the individual in 18,510 examined does; compound feed, particularly the protein-energy ratio, did not affect CM [18]. Analyzing data by age, there was a tendency for PCM to increase with the age of females, even though mastitic does were culled from first parities [8]. The agent determinants of mastitis might be part of the ubiquitous microbiota on the body or in the habitat, such as *Staphylococcus aureus* [19], *Pasteurella multocida*, or *Enterobacteriaceae*.

*Staphylococcus aureus* was the main agent in mastitic does: 65 and 67% of cases studied during 1970–1989, according to Renault and Maire, cited by [8]. It was 83.5% during 1990–1997, as pointed out by Baselga et al., *op. cit*. [8], and 71.2–74.0% more recently [9]. *Pasteurella multocida* was the main agent in 21.5%, 7.7%, and 13.9–15.3% of cases, respectively. Most conventional rabbits are carriers of low virulence *S. aureus* [20], able to infect the mammary gland. The diffusion of highly pathogenic strains of *S. aureus* between and within farms has worsened the problem [21].

Concerning the use of on-farm antimicrobials, Goñi et al. [22], and Ortega et al. [23] studied their efficacy against *S. aureus* diseases on commercial farms in Spain. According to the World Organization for Animal Health/OIE [24] and EFSA [25], restricted antimicrobial usage is unavoidable. In relation to the recent use of antimicrobials against mastitis, we had a knowledge gap and decided to focus their parenteral use in Spain.

The aims of this study were to (1) assess prevalence of clinical mastitis in female rabbits from January 2001 through March 2017 on commercial farms; (2) investigate farm-specific risk factors in relation to mastitis; (3) estimate some concurrent diseases other than mastitis during lactation; and (4) describe the on-farm use of parenteral antimicrobials against mastitis during 2012–2017.

## 2. Materials and Methods

Our study population comprised 1 January 2001 to 31 March 2017. We obtained our information by carrying out 2367 visits, as a part of veterinary practice, routine health appraisal, and consulting activities on rabbit farms. Animal Care and Use Committee approval was not obtained for this study because data were acquired from commercial farms that fulfill European, Spanish, and regional recommendations and laws on animal welfare, food safety, and environment protection [26].

### 2.1. Rabbit Farms, Doe Censuses, Breeds, and Lines

In this cross-sectional study, there were 531 farms with examined females; 490 were in Spain and 41 in Portugal. The 490 rabbitries were nationwide, located in 40/50 provinces in Spain. Rabbit meat farms were registered in the *REGA* (*Registro general de explotaciones ganaderas*), which is the official database of farms in Spain [26]. During July 2017, there were 906 rabbit farms housing >20 females, with a mean size of 713 does in Spain. In Portugal, there were 139 intensive farms, with a mean size of 842 does [27]. In the present study, to avoid bias with the rabbit censuses, on each visit, we asked producers about their rabbit doe inventories, i.e., females serviced once or more, generally at ≥4.5 months old. In relation to *rabbit breeds and lines*, we recorded those corresponding to the male and doe. In Spain and Portugal, the majority of food-producing rabbits belong to trademarks, with the exception of New Zealand White/NZW farms (we examined does on 2 farms for the laboratory and 2 for meat), and also farms with non-selected “colored” breeds/lines [28]. If there were >2 types on the farm, or non-determined lines, we classified them as “ND-2more”.

### 2.2. On-Farm Management Systems and Biosecurity Practices

In our practice, we visited farms with several production factors: open air and indoor farms, various trademarks and types of feed [18], rabbit lines, and management practices, described later. We recorded the type of service used on each farm: mount or insemination (AI), and day: 11-18-25-32-39-45 or 56 days after parturition (no ranges were allowed). In addition, we made a note of the number of batches per maternity barn: from 1 to 8; a batch is a group of rabbit does serviced on the same day. A farm with 1 batch, and AI on day 11 postpartum, had parturitions every 42 days, i.e., parities 8 times per year (or 8 batches yearly); however, the median kindling interval per doe was 51.4 days, i.e., 7.10 parturitions per doe per year [14]. Lastly, we recorded the antimicrobials used against mastitis; producers normally have this information available. With this database we formed a file of the general traits of the basic 531 farms to be appraised.

This study used cohort-level data. There were 2635 cohorts, i.e., groups of females organized by parturition day, breed or line. There were several female cohorts on each farm, depending on the *number of batches* or *lines* used. For instance, in a single-batch in a maternity barn, there might be (a) females only pregnant at their first service (b) females simultaneously lactating and pregnant, and (c) females only lactating, empty after the service; in this simplest case, there was 1 cohort at risk (b + c groups), i.e., the lactating does, our target in this study. In another example, there could be 1 batch of does, i.e., serviced on the same day, and belonging to 2 lines; in this case, we examined does arranged in 2 cohorts. In the *duo system*, all females were moved for parturition to clean disinfected barns with equipment in similar conditions. There were farms with 1 batch and *duo*, and farms with 1 batch without *duo* because the does remained in the same barn until they were culled or died. Farms with ≥2 batches per maternity barn did not have a *duo system*, but some farms had ≥2 maternity barns, 1 batch per barn, and a *duo system* in each one. 

### 2.3. On-Farm Diagnostic Work-Up

Clinical records used, herein, were carried out by a single, trained veterinarian (Rosell); however, all the 531 study farms were visited by several veterinarians, working for their rabbit producers’ associations, for feed suppliers, or laboratories, amongst others. In this study, the protocol for on-farm monitoring and surveillance of coryza, mastitis, ulcerative pododermatitis, and manges, included the examination of a sample of lactating does. For instance, with a known *n* of does to be examined in each cohort (Section 2.5), first we checked 10 to 15% of primiparous does (mainly, when they were grouped according to parity), which corresponded to the mean percentages of new does per batch [29]. We then proceeded with the multiparous does with a random systematic sampling; e.g., if *n* = 60, we examined 8 primiparous in a cohort with 550 females at risk; for the remaining 52 we examined 1 doe of 10, i.e., in total: 60 examined/550 does. In some cases, study farms were sampled for 2 consecutive months; therefore, a keeping-track procedure was applied to avoid repetitions of does. In our database, we have not included checked cohorts where does were culled *prematurely*, e.g., due to CM, nor check-ups with only first parity does. These assessments were supported by the physical examination of lactating does, and were done outside their housing, directly by visual examinations of clinical signs and gross lesions, or by palpation. In the clinical diagnosis of coryza, we checked the nose and medial surface of the forelimbs for coryza. We then examined the entire ventral region for CM, pyoderma, subcutaneous abscesses, mammae engorgement, or umbilical hernia, for differential diagnostic patterns [30]. We detected and diagnosed clinical mastitis by palpation of intramammary swelling, indurations, abscesses, or penetrating wounds of the mammary tissue. Then, we examined the skin covering the limbs, for ulceration, i.e., ulcerative pododermatitis, following our previously shown criteria [31], and psoroptic mange, sarcoptic mange, or both. All these criteria were classified as binary variables. Data concerning cohort traits and clinical outcomes were recorded at once during the on-farm examinations. Disease occurrence reported in this study was determined by evaluating animal-level prevalence [32]. In addition, we measured the kappa index of concordance of clinical diagnoses for mastitis; e.g., in January 2017, we examined 110 lactating does on a farm with 1200 does (and high PCM), sorted in 2 batches, and served with AI at the 25th day postpartum. We checked the same does with in a blind examination, three times on alternating days, 14–16 and 18 days postpartum.

### 2.4. Types of Visits by the Rabbit Veterinary Practitioner to Study Farms

From January 2001 through March 2017, data were gathered during our visits to farms, and did not follow an optimally balanced design; after the on-farm diagnoses, several visits were “emergencies”, and others were classified as “healthy” or “check-up”. A cohort with a result of PCM ≥10% was considered as (1) a case of “mastitis”, or (2) “staphylococcosis”, i.e., lesions compatible with these diseases. Lesions related with staphylococcosis were, e.g., (a) pustular pyoderma in does, in newborn and suckling rabbits, a pathognomonic lesion [33]; (b) ulcerative pododermatitis in young does in farms with footrests; (c) ulcerated forelimbs, digital dermatitis and necroses in does; (d) purulent (weepy) eyes, abscesses or both, mainly in kits; (e) even septicemia [34]. When we found high PCM and the cited lesions, we classified the case as “staphylococcosis”; however, there were sampled farms with high PCM and without other lesions compatible with *S. aureus* diseases. Images of these findings are available on our website: http://cunivetservice.com/docs/Poster.Rabbit.Mastitis%20and%20Staphyloc.June.15.2017.pdf. Each examined cohort with high PCM belonged to the following categories: “mastitis”, “staphylococcosis”, or “poor sanitary status”. On other visits, the main problem in an examined cohort was “ulcerative pododermatitis” (UP) (prevalence of UP (PUP) >10%), “poor sanitary status”: i.e., “respiratory” (P of coryza > 25%), besides PUP > 10% and PCM > 10%, with or without manges. Lastly, there were “healthy” cohorts.

### 2.5. Statistical Analysis

Sample size (*n*) calculation was done with *WinEpi* software [35], using the following data: population at risk (*n_l_* lactating does), degree of expected confidence (95%), and expected prevalence (*p*%); when the examination was made, we calculated apparent prevalence with the population at risk, sample examined (*n_e_* does), sick does found (*n_s_*), and degree of expected confidence (95%). We converted our anonymized raw data to Microsoft Excel 2007 (Microsoft Corp., Redmond, WA, USA). Statistical analysis was also by SAS [36], utilizing different GENMOD, UNIVARIATE, FREQ procedures, depending on the analyses we used. Statistical significance was indicated by a *p*-value < 0.05. The dependent variable: *prevalence of clinical mastitis* (PCM) was binomial (proportion) distribution in the GENMOD procedure. The units of analysis were the cohorts, (proportion: *n_s_* sick does/*n_l_* at risk). The factors of variation on the dependent variable (PCM) were estimated with the following model:

Y_ijklmnop_ = μ + A*_i_* + S*_j_* + C*_k_* + B*_l_* + D*_m_* + R*_n_* +W*_o_* + L*_p_* + e_ijklmnopq_

where μ was the population mean, A*_i_* was explained by the effect of the *i*th *year* (17 levels), S*_j_* was explained by the effect of the *j*th *season* (4 levels); C*_k_*, the effect of the *k*th *cause of visit* (2 levels: “emergency” vs. “healthy”); B*_l_*, the effect of the *l*th *number of batches* (3 levels: 1, 2, ≥3 batches per maternity barn); D*_m_*, the effect of the *m*th existence of *duo-system* (2 levels), R*_n_*, the effect of the *n*th *day of service*, (4 levels: 11, 18, 25, ≥32 days), W*_o_*, the effect of the *o*th *week of lactation* (6 levels), L*_p_*, with the effect of the *p*th *line* of male and doe (18 levels), and e_ijklmnopq_ was the residual effect.

## 3. Results and Discussion

### 3.1. Descriptive Farm and Breed or Line Statistic

**Figure 1:**
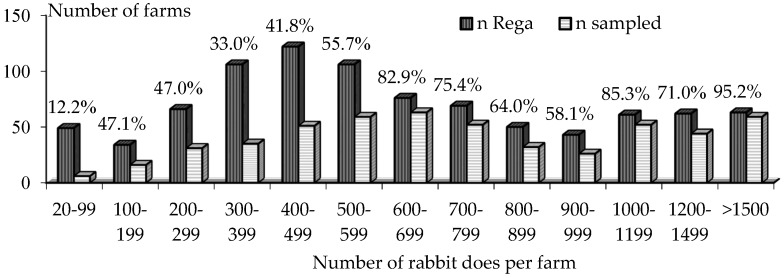
Size distribution of 906 rabbit farms registered in the official census of farms in Spain (*n* Rega), in summer 2017, and sizes of 490 study farms with examined does in Spain from 2001 to 2017 (*n* sampled). The percentages of sampled farms by strata are shown.

The raw data for this study was related to the 144,455 examined lactating females on 531 doe farms; 490 were in Spain and 41 in Portugal. Reporting data of 490 farms located in Spain were attributable to the specialized segment of farms housing >20 females, according to the *REGA* (Figure 1). The main demographic traits are given in Table 1. The majority of these large farms were for meat production; only 2 farms had 1000 and 275 female Rex rabbits. There were also 2 farms with 500 and 570 does, for laboratory rabbits, and 1 farm with 130 pet rabbit does.

Most farms also had weaned rabbits; in some cases, they were in the same barn, but frequently separated. Closed or indoor farms, i.e., not open air, had ≥1 maternity barn, and their does were in individual housing, without a platform, arranged either in the flat-deck or Californian way; we show images of collected examples on our website: http://cunivetservice.com/docs/Poster.Rabbit.Mastitis%20and%20Staphyloc.June.15.2017.pdf. On the 531 study farms, females were given complete commercial feed of several brands [18] and water ad lib. The 531 doe farms housed open breeding herds, i.e., introducing young does regularly, depending on their monthly culling and mortality risk of does; means were 7% and 3%, respectively [14]. Genetic types were described previously [28]. There are studies concerning genetic traits related with robustness of the rabbit, its ability to acquire alimentary resources and allocate them, with consequences on the lifespan of rabbit does or survival and weight of kits [37].

### 3.2. Farm Visits

A total of 2367 visits were carried out with 2635 cohorts, as we examined ≥2 cohorts on some farms; e.g., does of 2 lines served with their respective males, or does in ≥2 lactation stages. The median of the population sampled was 668 rabbit does per visited farm, (minimum to maximum: 83 to 6375 does), and the median size of the samples was 50 does, over 370 median does at risk per examined cohort (minimum to maximum: 40–3000 females). In relation to the frequencies of farm sampling, 245/531 farms (44.1%) were sampled once during the 17-year period, 93 farms twice, 57 three times, 15 four, 12 five, or 17 farms were sampled >25 times. From another perspective, there were 1012 single yearly samplings per farm; or 3 farms were yearly sampled >7 times.

In the present study, we found 720 cohorts with sanitary issues, over 2635 examined (27.32%). There were 321/720 cohorts with PUP > 10%, 176/720 cohorts with staphylococcosis as the main issue, 155 cohorts with mastitis, or 68 cohorts in poor sanitary status (Table 2).

Mean PCM was significantly different with *t*-tests among cohorts with “mastitis”, and cohorts classified as “healthy” (*t* = 2.07, *p* < 0.05). The classifications were made after the doe had been examined in each cohort. Currently, we are interested in studying reciprocal actions between mastitis and diseases other than mastitis, e.g., ulcerative pododermatitis, in the same doe; *S. aureus* is an opportunistic common agent in both issues [38]. A skin lesion can enable *S. aureus* to spread inside the organism, besides its pathogenicity [20].

### 3.3. Diagnosis of Clinical Mastitis and Disease Traits in Rabbit Does

Concerning on-farm clinical diagnosis of mastitic does, there were differences in relation to diagnosis in females of other animal species [39]. A crossbred young rabbit doe aged 4.5 months was priced at 15–30 euro. However, the residual value of a saleable doe was 0.5–1.0 euro at the abattoir. This was the main reason why the diagnosis was based on clinical signs of the abnormal gland (swelling, indurations, abscesses, skin ulcers, and open wounds), performed by palpation. In some cases, we observed signs in the abnormal doe (loss of condition, posture, decreased appetite, pain, fever), in agreement with Constable et al. [40]. After a presumptive diagnosis of mastitis, we did not use complementary methods (e.g., changes in milk characteristics). We necropsied some does, e.g., 134 from 2006 to 2014 (see below), and sent pus samples or expressed milk to the laboratory, for culture and drug susceptibility testing, as suggested by Meredith and Flecknell [41]. In the course of the study period, we observed one farm, (the 9 March 2011 case), with 650 does managed in a single batch, and 0.5% to 1% of females from first pregnancy, affected by general oedema, and mammary gland engorgement, in several batches. Pathologists from the *Universitat Autònoma de Barcelona,* diagnosed fibroadenomatous mammary hyperplasia; no attributable cause was found. 

To evaluate our clinical procedures for monitoring of mastitis, we determined concordance on a 1200 doe farm with high PCM. This was examined during January 2017 (Section 2.3). Determined apparent PCM in the 14th–16th and 18th days of lactation, was 22%, in 110 daily examined does. The Kappa index of concordance between the 14th and 16th day was *r* = 0.95 and 16 vs 18th, *r* = 0.96; between the 14th and 18th day, the Kappa index was 0.90. The lower concordance between the 14th and 18th days might be due to new cases of CM, and poorer accuracy in diagnosis. 

In this study, the majority of the 5992 does found with mastitis were chronic cases; there were 60–70% with abscess formation [42], followed by intramammary indurations, probably due to the clinical stage. We found few peracute cases, due to the lower incidence and higher mortality vs mild cases; during 2006–2014, we necropsied 134 does with mastitis, mainly acute forms, in the course of 3278 visits to 505 farms [13]. In our study, chronic mastitis were the most common forms; based on this, we cannot currently consider “blue breast” (or “red-black”), as synonymous of “mastitis”, as pointed out by several authors [43]. In this study, lesions in live females were mainly located in 1–2 mammae. According to Flatt et al. [44], “the inflammation quickly spreads from breast to breast until all breasts are affected”. On this matter, in a sample of 519 does with mastitis, 81% had one affected mammary gland and 15.3% had two [8]. Viana et al. [45] examined 130 does with chronic mastitis on several farms; 79.23% had 1–2 affected mammae.

### 3.4. Prevalence of Clinical Mastitis

In this study, we measured prevalence. During 2001–2017, the mean PCM determined retrospectively from 2635 cohorts and 144,455 examined lactating does on 531 farms, was 4.05% (CI_95%_ [3.87–4.22]); the within-farm PCM varied notably between farms (minimum to maximum: 0–36.00% PCM). We observed mastitis in almost all the sampled farms; throughout the study period, we sampled 193/531 farms ≥3 times. We did not come across diseased does with mastitis, only on 1 farm/193, over 5 samplings, with 148 examined does, and 415 does at risk. At the opposite end of the scale, PCM = 36.00% was a severe result. Some causes were found in high PCM, such as the lack of a global sanitary approach in practice; e.g., producers did not detect sick does before service, at pregnancy check, or at weaning. Another case was the absence of regularly scheduled health care services by veterinary practitioners, with monitoring and surveillance of CM. In such cases, there was a low culling risk of diseased does, thereby enabling on-farm transmission of *S. aureus* [20], as well as the disease spreading from the mammae to other organs, e.g., the uterus; these were main reasons for culling mastitic does [46]. Moreover, fostering kits from sick does was bad practice on some farms, as it enabled contagion [2]. In the present study, we observed the worst consequences of mastitis epidemics: closing farms due to endemic outbreaks of acute mastitis and multidrug-resistant *S. aureus*, which has also occurred in other countries [47,48].

From the year 1979 onwards, we have carried out physical examinations on 275,000 lactating does. The observed mean PCM in the 1970s and 1980s, in Spain, was >10%. Later, PCM was 5.2% in 1996 and 4.4% in 2000 [49]. In our opinion, PCM in domestic rabbits has decreased significantly. Many people and research centers worldwide have invested effort and input into the control of *S. aureus* diseases, the main cause of mastitis, focusing on the rabbit or other animals, including humans [50]. Based on this, we suggest a threshold for mastitis risk in rabbit females at 4% mean prevalence. It is evident that every rabbit farm should have its own target.

### 3.5. Risk Factors for Mastitis in Rabbits

In this study, PCM was influenced by several biosecurity variables, e.g., *duo system*, management practices, e.g., *number of batches* per maternity barn, also host factors (*breed* or *line*), and environmental risk factors (*seasonal effect*), as shown in Table 3.

The leading independent variable was *cause of visit*. Other explanatory factors were *day of service*, *lactation stage*, or *year*. Besides the test of significance of the effects of factors of variation for PCM, the odds ratio was determined for some variables (Section 3.5.3).

#### 3.5.1. Cause of Visit

In the present study, *cause of visit* was the leading independent variable (χ^2^ = 1144.78, *p* < 0.0001), in relation with PCM. It is noteworthy that classifications were made after the result of clinical examinations. The mean PCM of cohorts where “mastitis” was the most frequent disease was 13.27% (CI_95%_ [12.93–13.61]); this was significantly different from cohorts classified with “good health”: PCM = 2.71 (CI_95%_ [2.58–2.83]), with *t* = 2.07 (*p* < 0.05). “Ulcerative pododermatitis”, “poor sanitary status” or “staphylococcosis” were not significantly related (Table 2).

#### 3.5.2. Management Variables: “Type of Service” and “Day of Service” after Parturition

We found no differences between AI and mount for PCM. Insemination (AI) was used on 82% of the 531 farms (87% during 2012–2017); there were 2059/2635 examined cohorts with inseminated does, and 576 mounted. Our study suggests that the *day of service* had an effect (χ^2^ = 30.40, *p* < 0.0001). Prevalence of CM at 32–56 days of service = 2.55%, against the rest of the rhythms, with PCM = 4.06% (11th day), 4.23% (25th) and 4.36% (18th), although the length of the period of risk for CM varied considerably: from 65 days (e.g., does served 45 days postpartum) to 35 days (service at 11 days). During 2001–2017, females were served at 11 days postpartum on 76% of the farms, followed by the 18-day rhythm (10%), day 25 (9%), or ≥32 days (5%). These are key aspects, because the rhythm of reproduction and age at weaning, influence the degree of stress and health of does [51].

#### 3.5.3. Biosecurity: Effect of the “Duo System” on Maternities

There were 564 cohorts with *duo*, 1682 without, and 389 not determined. Mean PCM with the *duo system* was = 2.95% (CI_95%_ [2.81–3.09]), and without: PCM = 4.24% (CI_95%_ [4.12–4.35]). Differences were significant (χ^2^ = 37.83, *p* < 0.0001); additionally, the odds ratio was 1.55. Therefore, we may also consider this biosecurity practice as a useful tool; and reasonably, this improvement might be due to the decrease in infectious pressure.

#### 3.5.4. Effect of the “Lactation Stage”

With regard to PCM, there were significant differences between the *stages of lactation* (χ^2^ = 35.15, *p* < 0.0001). Prevalence of CM increased from 2.86% in the 1st week, to 4.36% in the 5th. Later on, PCM decreased moderately in the following weeks, to 4.33% in the 6th. One explanation for the observed peak in the 5th week is that on rabbit farms in Spain the 42-day cycle, i.e., AI on day 11 post-kindling, was followed by 76% of farms, with weaning at 35 d; diseased does were culled at this stage. In a previous study, we examined 15,700 does with a known age; CM showed a flat prevalence depending on the number of parities [18]. A next step should be to study a larger sample, if CM is age-related; this is an ongoing project.

#### 3.5.5. Effect of “Breeds or Lines” for Mastitis

Our study suggests that the host was a predisposing risk factor of disease. Most of the 531 study farms (96.2%) used crossbred synthetic lines traded by diverse European companies; for this reason, we describe the results for the sanitary status of the lines in little detail. There was an effect among >18 types of lines, breeds, and crossbreeds observed in this study (χ^2^ = 145.57, *p* < 0.0001). We obtained four groupings of significance: group A, 1 line with mean PCM = 7.09%, group B, 6 lines with PCM between 4.58% and 6.48%, group C with 6 lines and PCM between 3.54% and 3.96%, and group D with 4 lines and PCM between 1.29% and 3.24%. In a previous study during 2001–2003 [48], we compared the influence of the line of does on PCM, but only on farms where producers did not apply antimicrobials at kindling. Differences amongst PCM were also significant in such a study: PCM = 2.05% in the lowest, and PCM = 4.83% in the line most likely to have mastitis (*p* < 0.05). Perhaps with an individual analysis of the sanitary status we would know whether parity order and rabbit line are host influential factors for CM, directly, or due to a variety of intercurrent diseases. Rabbit producers replace their breeding stock, introducing in each batch young females (grandparents or crossbred does), semen, or both, purchasing from AI centers or breeding companies. Therefore, *S. aureus* carriage among males [52], and females [53], is a key aspect in rabbit health assessment.

#### 3.5.6. Biosecurity Variables: Effect of the “Number of Batches” per Maternity Barn

On the 531 sampled farms, there were 1187 cohorts with 1 single batch, 428 with two, 910 from 3 to 8 batches, and 108 not determined. Mean PCM = 3.24% (CI_95%_ [3.13–3.45]) with 1 batch, and 4.07% (CI_95%_ [3.81–4.25]) with 2; main significant differences were for ≥3 batches: 5.10% (CI_95%_ [4.92–5.28]), (χ^2^ = 47.08, *p* < 0.0001). Reducing the number of batches, and avoiding contact between does in different stages of lactation, was an effective biosecurity measure to prevent disease. 

#### 3.5.7. Effect of Environmental Risk Factors: “Seasonal Effect”

In this study, we found *seasonal effects* on PCM in does (χ^2^ = 42.37, *p* < 0.0001). The overall mean PCM during spring (April to June in the Iberian Peninsula) was higher: 4.51%, followed by winter: PCM = 4.11%, autumn: PCM = 4.04%, and summer: PCM = 3.60%. From the climatic perspective, we visited open-air farms, indoor farms with static ventilation, and rabbitries with dynamic ventilation by extractors (Section 3.1); from the viewpoint of this production factor, some farms were well managed, while others were not (this was an enabling risk factor). To be certain of this effect, we should compare farms with or without ventilation systems to compensate the outdoor climate.

#### 3.5.8. “Year Effect”

**Figure 2:**
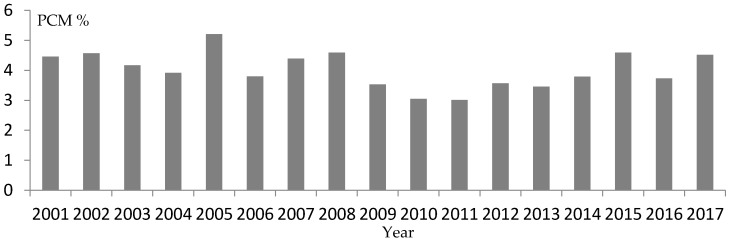
Yearly prevalence of clinical mastitis (PCM %), with physical examination of 144,455 lactating rabbit does on 490 rabbitries, in Spain, and 41 in Portugal.

We analyzed the yearly PCM. There were significant changes amongst years (χ^2^ = 97.00, *p* < 0.0001). However, there were few important differences, because there was no uniform tendency during the 17-year study (Figure 2).

From 1986 (PCM = 9.1%) to 1996 (5.2%) the year had an enabling effect [8]; however, PCM was 4.40% during 1997–2000 [48], and 4.05% during 2001–2017.

### 3.6. Use of Antimicrobials

The present study reports the therapeutic practices against mastitis in rabbits. From a subset of 200 rabbit farms, visited from 1 January 2012 to 31 March 2017, we determined that 140/200 (70%) of producers administered antibiotics systemically (mainly: subcutaneously/sc) at kindling in first parity, or even in multiparous females. The median size of these farms was 834 does (minimum to maximum: 100–6375 does), and the median of the sampled cohorts 400 does at risk (minimum to maximum: 50–1640 does). On farms where producers did not inject their females, median sizes were 750 does and 320 does, respectively. Females were injected sc 4–5 days before parity, mainly 4–5 days after, or both. The delay after parity was principally recommended in the case of β-lactam antibiotics, to avoid adverse effects in the doe (gut microbiota dysbiosis) or to her litter, when administered on the day of parturition.

In this retrospective study, we found several treatment patterns. On the 140 farms where all the primiparous, multiparous does, or both, were injected sc at parturition, 119/140 (85%) were administered β-lactam antibiotics. On 100/119 (84%) of these farms, does were injected sc with a single dose of penicillin G (PG) benzathine (15,000 to 30,000 IU/kg BW), plus PG procaine (dose: *idem*), including streptomycin in some compounds (45 mg/kg BW). On 19/119 (16%) of the farms using β-lactam antibiotics, producers applied long-acting amoxicillin (15–30 mg/kg BW); single dose sc, sometimes repeated 48 h later. According to the producers, both antibiotics were prescribed to control *S. aureus*, for metaphylaxis use in at-risk does, for acutely diseased, or chronically mastitic does. In 21/140 (15%) of the farms using injections, the prescribed antimicrobials were macrolides, or long-acting oxytetracycline, to also control *S. aureus* and *P. multocida*.

Our previous findings were the following: on 35% of 250 visited commercial rabbit farms during 1992 to 1994, producers applied antibiotics at kindling; in 72% of cases, they used penicillin compounds [54]. Throughout the year 2000, on 54% of the visited farms, does were antibiotic-treated, and with 82% penicillin [55]. Penicillin was the treatment of choice against mastitis, as well as management practices: e.g., “protection from changes *in* temperature” [11]. Drug dosages recommended were, for PG benzathine: 42,000–60,000 IU/kg q48h sc, im, or PG procaine: 42,000–60,000 IU/kg q24h sc, im [30].

In our practice, we focus on the treatment for the effectiveness and adverse reactions; e.g., *severe*: enteritis and death or *mild*: enteritis and infertility. For instance, we have observed farms from 1 to 5% treated does having enteritis-diarrhea and death, with a single sc dose of 15,000 IU PG procaine plus 15,000 IU of PG benzathine/kg BW. On the other hand, there were mild or no reactions, on farms where producers administered one sc dose with 30,000 IU PG procaine form +30,000 IU PG benzathine/kg BW; similar cases occurred with amoxicillin. Results of several cecal samples’ content submitted for microbiological analysis, showed mainly presence of *Escherichia coli*, *Clostridium spiroforme*, or both. They were communicable to the AEMPS, the Spanish Agency of Medicines and Medical Devices. Antimicrobials for parenteral administration were not labeled for rabbits, and this was the main reason why culled does were non-saleable for consumption, due to long withdrawal periods (≥28 d), and were destined for rendering; in fact, penicillin was considered a major claim for rabbits, years ago [56]. In France, all those working in the rabbit industry initiated measures to decrease the use of antibiotics [57]. To begin with (2009), the use of injections, in a sample of 95 nationwide visited farms, was on 20/95 farms (21%), mainly in females [58], and 8% of the total use of antimicrobials, in relation to kg BW treated; it included cephalosporins (“with moderate use”), amongst others, but excluded penicillin and amoxicillin, “toxic for the species” [57]. 

The next step on future farm health visits will be to encourage producers to change their practices, e.g., controlling misapplication [59]. Alternatives should include (a) the stimulation of the immune system; also (b) a better knowledge of the microbiota of the rabbit mammary gland, including *S. aureus*; (c) a better knowledge of the host’s resilience traits; and (d) their interactions with the environment [60], or the risk factors for dysbacteriosis. In agreement with Ritter et al., [61], rabbit producers need clear data and motivation. The goal of our on-rabbit farm CM control program is to palliate the severity of outbreaks, (a) by reducing the degree of misuse of antibiotics; (b) by increasing the number of laboratory analyses; and (c) implementing biosecurity measures, some of which were determined in the present study. It is a reachable objective, because our results show that the attitude of producers towards rabbit welfare is good, in accordance with Garforth [62]. 

### 3.7. Limitations of the Study

This study was based on a convenience sample of farms that we had access to, from 2001 to 2017. We probably cannot extrapolate our results to the larger populations: 490 sampled farms vs 906 registered farms, and 41 vs 139, in Spain and Portugal, respectively. In function, in our work protocol, *low* sampling farms’ misclassification occurred sometimes because (a) we avoided examining does during the week after the service; and (b) exceptionally, we declined to examine farms with frightened does that were thumping the housing floors. In Section 2.3 and Section 3.3 we presented methodology and results, and discussed our diagnostic tools available to conduct a field study; it was impractical to analyze (e.g., bacteriological), all presumptive cases of CM diagnosed on rabbit farms. We determined the Kappa index of concordance of clinical diagnosis, done by the same person. Concerning differential diagnosis, e.g., of mammary tumors [63], the percentage of clinically and alive examined does affected should be low; according to on-farm post-mortem observations [13,14], does on commercial farms were young. Cases of mammary gland engorgement were discussed previously in Section 3.3.

In relation to confounding evaluated explanatory variables, there was a pool of threats probably influencing rabbit stress and health, but we have not described the majority of them; for instance, (a) the degree of coping with climatic variables in their habitats, and mainly the triad: temperature, humidity, and air speed, due to micro-changes in weather. We have not measured (b) the monthly doe replacement risk, (c) hygiene practices in each barn, (d) other practices, e.g., the mother–litter separation on the first 11 days postpartum [64] or the number of siblings per litter, (e) feeding troubles and outbreaks of gastroenteritis, or (f) other underlying immunosuppressors that might be correlated, e.g., myxomatosis, and their vaccinations [65]. We have not analyzed (g) other variables common to livestock, such as dry period feeding or some intercurrent diseases [66]. Lastly, some biosecurity measures were not included in this study, e.g., (h) origin of young females, (i) quarantine practices, (j) cleaning and disinfection schedules, or (k) degree of training of producers, amongst other risk factors [67].

Concerning the model used in the statistical analysis, we included all the available factors recorded during visits to farms. The analysis was done using a forward elimination procedure, e.g., for the geographical variables; and also backward, such as *type of service* (Section 3.5.2). Reciprocal actions between several factors were evaluated; e.g., *day of service* and *stage of lactation* had 28 groups of interaction, some with low *n*; e.g., *n* = 4 for AI 32 days after parity × 1st lactation week. Another possible confounder was “on-farm therapeutic practices”. For instance, there were 1350 cohorts where does received a sc dose of antibiotic near parturition, with PCM = 4.04 ± 3.94%. There were 912 cohorts without treated does (PCM = 4.53 ± 5.03%); the results do not differ significantly with *t*-test = 0.19 (*p* > 0.8). In addition, we only determined prevalence, because the incidence risk of mastitis was measured just on some hundreds of does, and consequently, this source of information might be biased [68]. Finally, with reference to the recorded use of antibiotics, we have chosen the last 200 visited farms, suitable to describe the most recent practices, on a sample of farms representing various types, sizes, and factors of production.

## 4. Conclusions

This paper deals with several epidemiological (prevalence, risk factors), and control aspects (diagnosis, prevention, and treatment), related with clinical mastitis (CM) on rabbit farms, during 2001–2017. The data suggest that prevalence of CM = 4% may be considered baseline on the sanitary scoring of female rabbits. However, on some rabbit farms, CM was a serious problem, due to a long history or to acute outbreaks; indeed, mastitis, staphylococcosis, or both, lead producers to close their farms. There were several predisposing (e.g., the line), and enabling (e.g., the season) risk factors for CM. Producers should receive reliable information on the adverse effect of certain management practices; e.g., it may be advisable to take action by reducing the number of batches per maternity barn. Besides, they should be motivated to invest resources in biosecurity; e.g., in relation to the use of a *duo system*, with cleaning and disinfection of the barns and their equipment before parturitions. In relation to the host, there were line differences in susceptibility to mastitis. Hence, these factors need to be taken into account to enhance the sustainability of rabbit production, including the prudent use of antimicrobials.

## Figures and Tables

**Table 1:** Overall descriptive traits of the lines of 144,455 rabbit females physically examined on 490 farms in Spain and 41 farms in Portugal from 2001 to 2017.

Trait	N Farms ^a^	Does	Q2 Does ^b^	Does at Risk	Q2 Does Risk ^b^	N Exams	Examined Does	Q2 of Samples
Colored	14	26,946	668	13,633	325	39	2394	50
Hyplus ^c^	93	256,098	815	142,632	430	301	17,677	50
Male Hyplus ^d^	50	113,105	970	61,273	438	110	6394	50
Hycat ^c^	10	11,215	770	5004	376	15	785	50
Hycole ^c^	10	13,349	625	6124	360	21	948	45
Hyla ^c^	18	72,486	600	38,182	450	64	3352	50
IRTA ^e^	30	81,888	680	26,722	272	70	4073	60
IRTA *Prat*	4	9817	210	6619	91	50	2883	51
IRTA *Caldes*	3	5046	150	2506	67	32	1903	30
Male IRTA ^d^	8	55,194	266	39,408	158	127	7840	41
ND-more2 ^f^	216	488,313	746	225,674	400	457	25,764	50
NZ White	4	11,804	511	7355	288	24	1280	40
Rex	2	9757	556	2586	143	16	810	50
UPV ^c^	208	775,196	680	457,957	400	983	55,844	50
UPV-V	21	95,616	590	67,756	260	208	9763	40
UPV-H	4	7048	780	1986	67	22	467	25
UPV-LP	7	7241	515	1833	50	24	581	21
UPV-R	8	21,271	353	5239	75	72	1697	24
Total	710	2,061,390	668	1,112,489	370	2635	144,455	50

^a^ N of visited farms was 531, not 710. This means that many farms changed their strain of rabbits in the course of the 17-year study period. ^b^ Median size (Q2) of the visited farms and Q2 of the size of the examined cohorts at risk, respectively. ^c^ On these farms, the does belonged to maternal lines and were served by males of the same line, selected for feed efficiency. ^d^ On these farms, the does belonged to several lines but were crossed with different males: PS-40, PS-119 (Hyplus) or IRTA-*Caldes*. ^e^ IRTA corresponds to crossbreed does of male UPV-V with IRTA Prat doe, served by male Caldes. ^f^ ND-more2 means farms with more than 2 lines with indeterminable crosses, or without available information.

**Table 2:** Prevalence of clinical mastitis and diseases other than mastitis in 2635 cohorts of rabbit does physically examined on 490 farms in Spain and 41 farms Portugal from 2001 to 2017.

Type of Visit	PCM ± SD ^a^	*n* Farms	*n* Visits	*n* Cohorts	Does at Risk (*n*)	Examined Does (*n*)	*n* Mastitis
Healthy ^b^	2.71 ± 2.86	303	1675	1915	777,259	103,324	2833
Mastitis ^c^	13.27 ± 4.23	86	153	155	74,736	8058	1057
PSS ^d^	11.37 ± 6.33	51	67	68	38,758	4751	512
Ulcerative podo ^e^	4.10 ± 3.73	190	306	321	136,672	16,884	717
Staphylococcosis ^f^	7.64 ± 6.53	107	166	176	85,064	11,438	873
Total	4.05 ± 0.09	737 ^g^	2367	2635	1,112,489	144,455	5992

^a^ PCM: prevalence of clinical mastitis (%) and standard deviation (SD). ^b^ Visits including physical examination of lactating does, with good sanitary status. ^c^ Cohorts with ≥10% of does with clinical mastitis. ^d^ Poor sanitary status (PSS): with prevalence of coryza ≥25%, PCM ≥ 10%, and prevalence of ulcerative pododermatitis ≥10%, at the same time. ^e^ Cohorts with ≥10% of does with ulcerative pododermatitis. ^f^ Examined does with pustular dermatitis; the females, the suckling kits, or both. ^g^ There were 531 farms, but during the 17-year period they were classified in different sanitary status.

**Table 3:** Factors of variation for prevalence of clinical mastitis of rabbit does, and test of significance of the effects. Physical examination of 144,455 does on 490 rabbit farms in Spain and 41 farms in Portugal, from 2001 to 2017.

Factor of Variation	Degrees of Freedom	Chi-Square	*p* > Chi
Cause of visit	1	1144.78	<0.0001
Day of service	3	30.40	<0.0001
Duo system	1	37.83	<0.0001
Lactation stage	6	35.15	<0.0001
Line or breed	17	145.57	<0.0001
Number of batches	2	47.08	<0.0001
Season	3	42.37	<0.0001
Year	16	97.00	<0.0001
